# Structure and number of mating pheromone genes is closely linked to sexual reproductive strategy in *Huntiella*

**DOI:** 10.1186/s12864-023-09355-9

**Published:** 2023-05-13

**Authors:** Andi M. Wilson, Michael J. Wingfield, Brenda D. Wingfield

**Affiliations:** Forestry and Agricultural Biotechnology Institute (FABI), Department of Biochemistry, Genetics and Microbiology, University of Pretoria, Pretoria, 0028 South Africa

**Keywords:** Sexual reproduction, Homothallism, Unisexuality, Heterothallism, Mating pheromones, *Huntiella*, Sexual strategy, Mating strategy

## Abstract

**Background:**

*Huntiella* resides in the Ceratocystidaceae, a family of fungi that accommodates important plant pathogens and insect-associated saprotrophs. Species in the genus have either heterothallic or unisexual (a form of homothallism) mating systems, providing an opportunity to investigate the genetic mechanisms that enable transitions between reproductive strategies in related species. Two newly sequenced *Huntiella* genomes are introduced in this study and comparative genomics and transcriptomics tools are used to investigate the differences between heterothallism and unisexuality across the genus.

**Results:**

Heterothallic species harbored up to seven copies of the a-factor pheromone, each of which possessed numerous mature peptide repeats. In comparison, unisexual *Huntiella* species had only two or three copies of this gene, each with fewer repeats. Similarly, while the heterothallic species expressed up to 12 copies of the mature α-factor pheromone, unisexual species had up to six copies. These significant differences imply that unisexual *Huntiella* species do not rely on a mating partner recognition system in the same way that heterothallic fungi do.

**Conclusion:**

While it is suspected that mating type-independent pheromone expression is the mechanism allowing for unisexual reproduction in *Huntiella* species, our results suggest that the transition to unisexuality may also have been associated with changes in the genes governing the pheromone pathway. While these results are specifically related to *Huntiella*, they provide clues leading to a better understanding of sexual reproduction and the fluidity of mating strategies in fungi more broadly.

**Supplementary Information:**

The online version contains supplementary material available at 10.1186/s12864-023-09355-9.

## Background

One of the most remarkable characteristics of fungi is that they are capable of sexual reproduction via numerous different strategies. For example, heterothallism describes the self-sterile system that requires an interaction between two compatible mating partners. In contrast, homothallism describes the self-fertile system that allows for sexual development within an individual derived from a single cell. Homothallism is an aggregate term that describes at least five different forms of behavior; primary homothallism, pseudohomothallism, bi- and uni-directional mating type switching, and unisexuality [1]. Transitions between these diverse sexual strategies are well-documented in ascomycete fungi, with closely related species sometimes exhibiting different sexual strategies [2–6]. In a number of notable cases, single species are able to utilise different sexual behaviors depending on the environment [7, 8].

The transition from one sexual strategy to another is often facilitated by the rearrangement of the mating-type (*MAT*) locus; a region of the genome that encodes proteins that regulate sexual development in most fungi (reviewed in [9]). In general, rearrangements that bring together the *MAT1-1-* and *MAT1-2-*associated genes result in homothallism [10]; while those that break up these genes result in heterothallic species [11]. For example, the ancestor of the genus *Neurospora* was likely heterothallic and genetic events, such as unequal crossing over, have resulted in different *MAT* locus structures that confer homothallism [6, 12]. Similarly, heterothallism may also be the ancestral state in *Aspergillus*, with duplication and transposition events likely having played a role in the transition to homothallism in species such as *Aspergillus fischeri* [13]. Comparisons of the *MAT* loci in heterothallic and homothallic fungi in the *Botryosphaeriales* [14], *Calonectria* [5], *Leptographium* [15], and *Thielaviopsis* [4] also support the existence of a heterothallic ancestor from which transitions to homothallism have occurred, sometimes multiple times.

While *MAT* locus rearrangement is often sufficient to bring about changes in the sexual behavior of a species [9], the transition between sexual strategies is also associated with changes in other processes. An example of this is the pheromone response cascade; a pathway that is essential for partner seeking in heterothallic fungi. Despite being maintained in a variety of self-fertile fungi, the genes associated with the pheromone response pathway are typically dispensable for sexual reproduction in these species [16, 17]. This suggests that the proteins encoded by these genes may have been co-opted for a different function, either related to mating or not [reviewed in [18]]. An interesting morphological change has also occurred in species that exhibit homothallism in *Neurospora*. While heterothallic *Neurospora* species produce abundant conidia, the fertilizing agents for outcrossing events, homothallic *Neurospora* species do not produce fertilizing cells of any kind [6]. Taken collectively, this suggests that during the transition to self-fertility, a variety of developmental pathways, both molecular and physiological, have been redirected for other purposes or entirely lost.

**Fig. 1:**
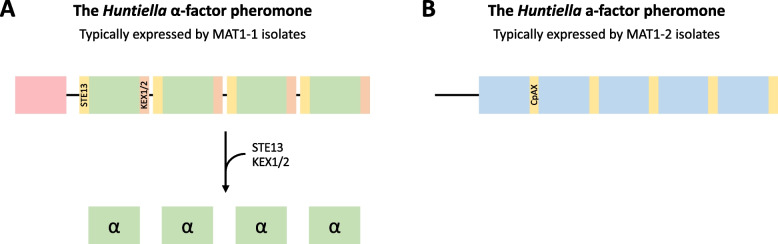
Structure of the *Huntiella* α- and a-factor pheromones. **A** The α-factor pheromone is initially translated as a large pre-protein, possessing a signal peptide (indicated in red) and harboring numerous repeats of the mature pheromone (indicated in green). This protein is subsequently cleaved by a variety of enzymes, including STE13 (cleavage site indicated in yellow), and KEX1/2 (cleavage site indicated in orange), which release the individual mature pheromone factors. The structure of the *Huntiella* α-factor is similar to the structure of the canonical ascomycete α-factor. **B** The *Huntiella* a-factor is translated as a short peptide and also harbors numerous repeats (indicated in blue), each ending in a conserved CpaX domain (indicated in yellow). The *Huntiella* a-factor is comparable to the a-factor from *Fusarium* [23] and the h-type pheromone from *Verticillium* and *Trichoderma* [24]. However, it is quite different from the canonical ascomycete a-factor, which typically possesses only a terminal CAAX domain. This terminal CAAX domain is modified during pheromone maturation. Given the unique structure of the *Huntiella* a-factor pheromone, it is unknown whether additional processing of this pheromone takes place

In ascomycete fungi, the pheromone response pathway is initiated when one of two mating pheromones binds its cognate receptor, activating a kinase cascade which ultimately leads to the differential expression of genes associated with mating [19]. In general, MAT1-1 isolates express the α-factor pheromone, a short pheromone that is expressed as a large pre-protein and is processed into a mature pheromone factor via rounds of cleavage by enzymes including STE13, KEX1, and KEX2 (Fig. 1) [19, 20]. This pheromone is recognized by the MAT1-2 partner via its interaction with the STE2 receptor. Similarly, the a-factor pheromone is expressed by MAT1-2 isolates (Fig. 1) and interacts with STE3 receptors expressed by the MAT1-1 partner. Alterations to mating type-dependent expression of these pheromones have been hypothesized to result in the transition from heterothallism to homothallism, particularly unisexuality, in *Candida albicans* [7], *Neurospora africana* [21], and *Huntiella moniliformis* [22].

The genus *Huntiella* accommodates just over 30 predominantly saprobic fungi within the Ceratocystidaceae [25–27]. While most *Huntiella* species are known to be heterothallic, at least two species, *H. moniliformis* and *Huntiella fecunda*, have been formally described as unisexual [28, 29]. The existence of two different sexual strategies within a group of closely related species, together with the availability of genomic and transcriptomic NGS data for a number of these species [22, 30–35], has provided the opportunity to investigate the genetics of self-fertility and self-sterility in *Huntiella*.

The aim of this study was thus to investigate whether the genetic patterns previously associated with unisexuality in *H. moniliformis* are present in two newly described unisexual *Huntiella* species*, H. fecunda* and *Huntiella tyalla*. By sequencing the genomes of these two additional species and comparing the *MAT* loci and mating pheromone genes in the three homothallic fungi to similar data for a diverse set of heterothallic *Huntiella* species, it was possible to identify putative genetic factors that are associated with the transition from heterothallism to homothallism in these species.

## Results

### Genome assemblies of *H. fecunda* and *H. tyalla*

The genome assembles of *H. fecunda* and *H. tyalla*, sequenced using the Illumina HiSeq platform and assembled using SPAdes v3.13.0 [36], were both approximately 25Mb in size, comparable to the other *Huntiella* genomes sequenced to date (Table S1). The *H. fecunda* genome was assembled into 357 contigs, of which 255 were larger than 1Kb (Table S2). The N50 and L50 values were 320 262 and 22, respectively and the GC content was 48%. The BUSCO analyses indicated that this genome was 98.0%, 96.0% and 86.9% complete with respect to the Fungi, Ascomycete and Sordariomycete databases, respectively. Similarly, the *H. tyalla* genome was assembled into 317 contigs, with 241 being larger than 1Kb (Table S2). The N50 and L50 values were 302 606 and 24, respectively and the GC content was 48%. The BUSCO analyses indicated that this genome was 98.3%, 96.0% and 86.9% complete with respect to the Fungi, Ascomycete and Sordariomycete databases, respectively.

**Fig. 2:**
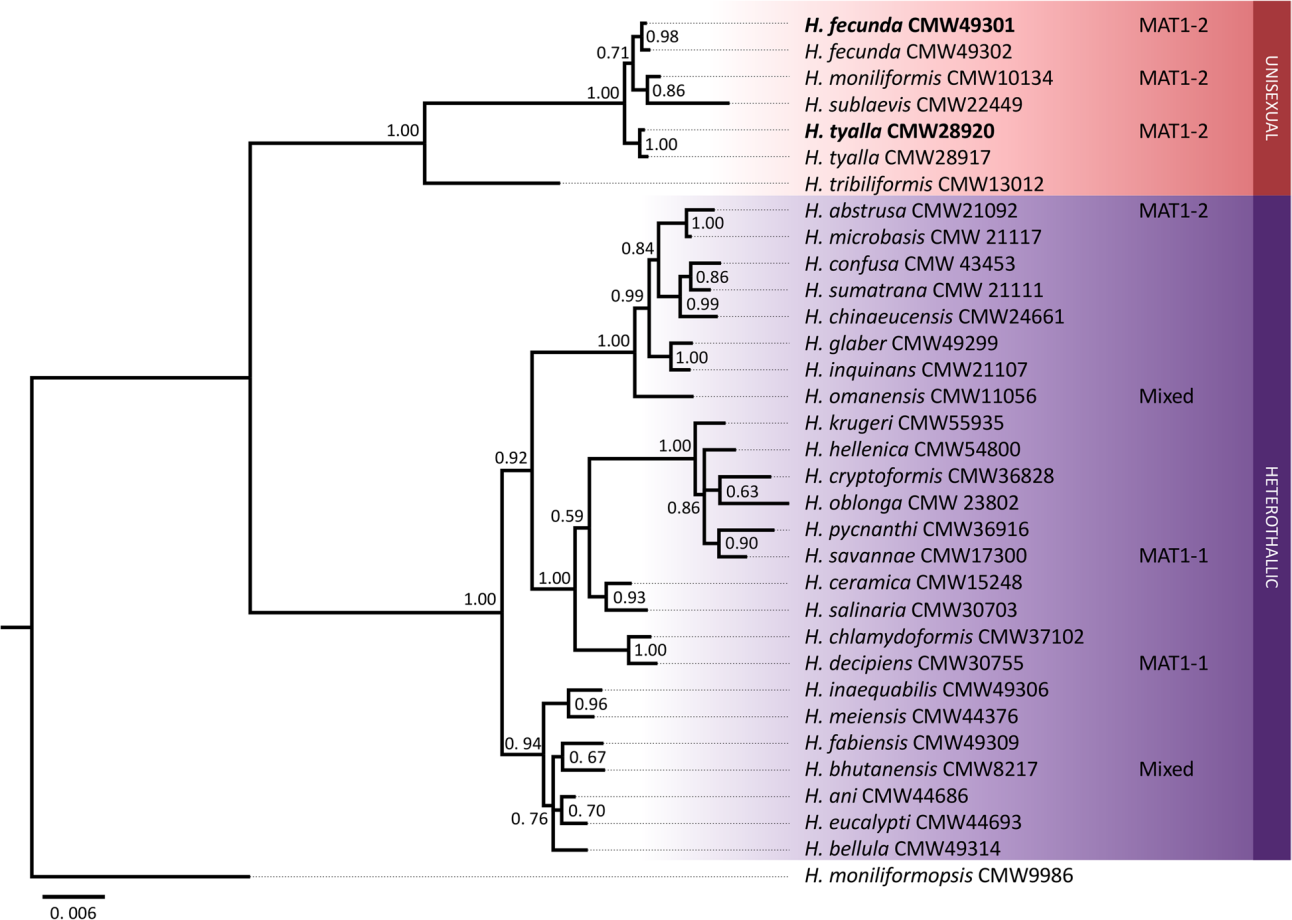
Phylogeny based on Bayesian analysis of three gene regions (*BT1*, *ITS*, and *TEF1*) from various *Huntiella* species. The two genome sequences generated for this study were from the isolates in bold text. The mating type of isolates for which genomes are available are indicated as MAT1-1, MAT1-2, or mixed. The red clade harbors species that are thought/known to be unisexual, while the purple clade harbors species thought/known to be heterothallic. Posterior probabilities are indicated at each node. *Huntiella moniliformopsis* was included as an outgroup

The phylogenetic analyses confirmed the identity of the isolates used for genome sequencing (Fig. 2). The sequences for the *H. fecunda* genome clustered with a second isolate of *H. fecunda* (CMW 49302) and sequences for the *H. tyalla* genome clustered with a second isolate of *H. tyalla* (CMW 28917). As expected, both were closely related to other species within the Indo-pacific clade of the genus, including *H. moniliformis* and *Huntiella sublaevis* [26, 28].

### *H. tyalla* is unisexual

In order to determine the sexual strategy of *H. tyalla* and confirm the unisexual ability of *H. fecunda*, single hyphal tip isolates were generated from both species. In both cases, these isolates were able to complete the sexual cycle in isolation when incubated under conditions conducive to sexual development in *Huntiella* species. Genomic analyses showed that both species harbored the *MAT1-2* idiomorph, while genes associated with the *MAT1-1* idiomorph were not identified from either of the genomes. This confirmed that *H. fecunda* is a unisexual species and indicated that *H. tyalla* is also capable of unisexual reproduction.

### The *MAT1-2-7* truncation is present only in unisexual *Huntiella* species

**Fig. 3:**
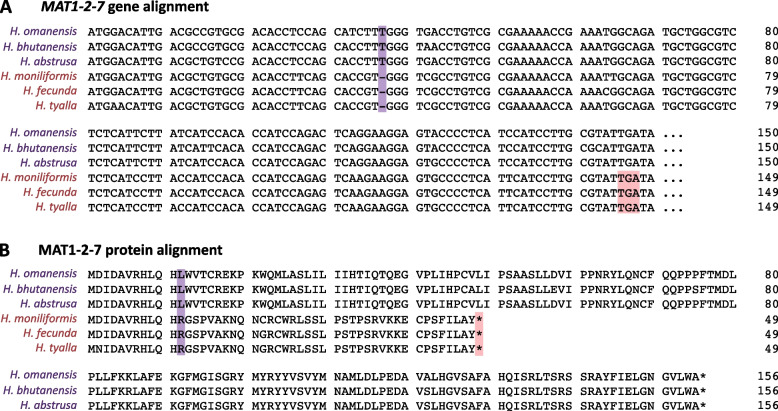
Alignments of the **A**) *MAT1-2-7* gene and **B**) MAT1-2-7 protein from heterothallic (purple) and unisexual (red) *Huntiella* species. A thymine residue at position 37 (purple boxes) was deleted in the *MAT1-2-7* gene from all three unisexual species. This mutation changed the reading frame of the gene and thereby introduced a premature stop codon at position 145 (red boxes). The six species included in this figure are the six species for which genomes representing the MAT1-2 mating type were available

The *MAT1-2-7* gene of *H. moniliformis* was previously shown to harbor a premature stop codon due to a single base deletion that induced a frameshift mutation. The premature stop codon truncates the would-be protein to only 18% of that in the heterothallic *H. omanensis* [29]. In the present study, the same single base deletion was found in *H. fecunda* and *H. tyalla* (Fig. 3). The deletion is supported by RNAseq reads that were mapped to this region in *H. moniliformis* to confirm that the deletion was not a sequencing or assembly error (Fig. S1).

This deletion changed the reading frame of the *MAT1-2-7* gene in all three unisexual species, thereby significantly altering the MAT1-2-7 protein sequence from amino acid position 12 onwards (Fig. 3). Consequently, this shortened and altered protein was most likely non-functional in all three unisexual *Huntiella* species, while being maintained in diverse heterothallic species, including *H. abstrusa*, *H. omanensis*, and *H. bhutanensis*.

### *Huntiella* species have multiple copies of the a-factor pheromone

**Fig. 4:**
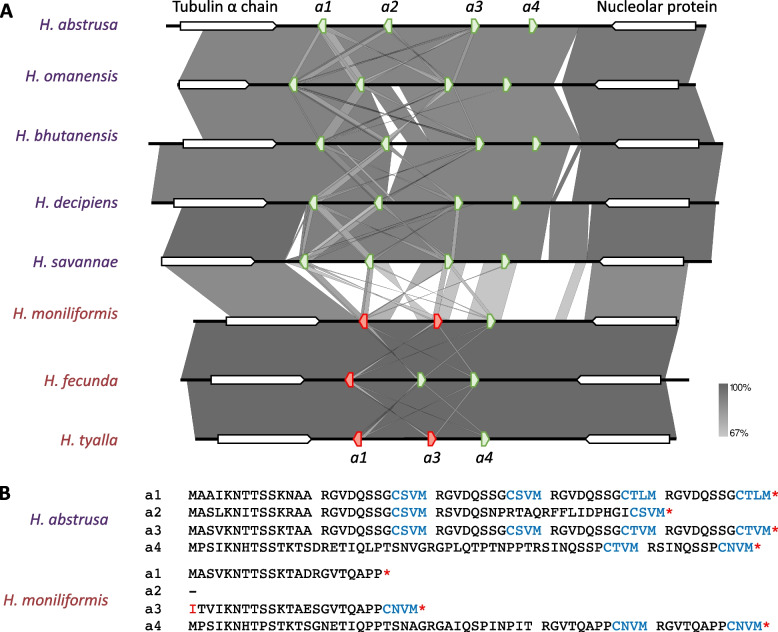
The first *Huntiella* a-factor pheromone locus, harboring four genes in the five heterothallic species and only three genes in the three unisexual species. These pheromone genes are flanked by genes encoding a tubulin α chain and a nucleolar protein. **A** A synteny map of this locus across all eight species considered, showing high levels of similarity within the heterothallic species and within the unisexual species. Significant regions of the heterothallic loci are missing from the unisexual loci, including one of the a-factor pheromone homologs. This figure was generated using EasyFig V2.2.2. **B** Protein sequences of the homologs from selected heterothallic and unisexual representatives. This shows the modular nature of these proteins, with the CpaX domain indicated in blue text and stop codons indicated by an asterisk. The *a4* gene is likely the only homolog that produces a full length and functional protein in *H. moniliformis*

**Fig. 5:**
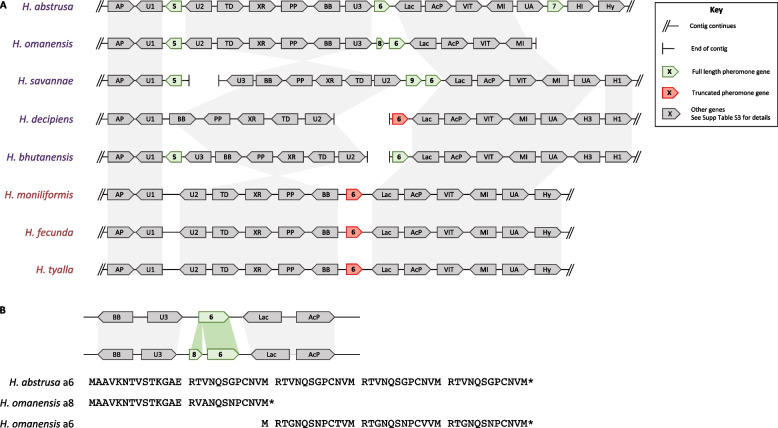
The second *Huntiella* a-factor pheromone locus, harboring up to three additional a-factor pheromone homologs. **A** Gene maps of the locus showing conservation of gene content with evidence for an inversion of the region between genes U2 and U3. This inversion appears to have occurred in the ancestor of *H. savannae*, *H. decipiens,* and *H. bhutanensis*, as the locus structure is shared by *H. abstrusa*, *H. omanensis* and the three unisexual species. Notably, *H. decipiens* and the three unisexual species lacked the *a5* genes. Additionally, their *a6* genes were truncated. This figure was drawn using data from BLASTn and tBLASTn analyses using the *H. abstrusa* homologs as queries and is not drawn to scale. The identity of the additional non-pheromone genes at this locus can be found in Table S3. **B** A zoomed in region of the gene maps from *H. abstrusa* and *H. omanensis* and the sequences from the *H. abstrusa* a6 protein and the *H. omanensis* a6 and a7 proteins. This shows that the gene is split in *H. omanensis*, with the *H. omanensis a8* gene encoding a protein homologous to the N-terminal of the *H. abstrusa* a6 protein and the *H. omanensis a6* gene encoding a protein homologous to the C-terminal of the *H. abstrusa* a6 protein

There were up to seven putative genes encoding the a-factor pheromone in the eight *Huntiella* species investigated in this study (Figs. 4 and 5).

#### The first *a*-factor pheromone locus

The first four putative a-factor pheromone genes, named *a1*, *a2*, *a3,* and *a4*, were flanked by genes encoding the tubulin α-chain and a nucleolar protein in all five heterothallic species and were arranged with *a1* and *a2* in the reverse orientation and *a3* and *a4* in the forward orientation (Fig. 4). In general, *a1* and *a3* encoded proteins that exhibited the most conserved structure, with multiple repeating units of the putative mature pheromone. These repeating units were 12 amino acids long, terminated in a conserved CpaX domain, and were almost identical (Fig. 4, Fig. S2). In contrast, despite possessing terminal CpaX domains, the proteins encoded by *a2* and *a4* typically harbored fewer recognizable repeats.

A similar a-factor pheromone locus was present in the three unisexual species, flanked by the tubulin α-chain and nucleolar protein genes (Fig. 4). However, only three pheromone-like gene regions could be found at this locus, with *a2* missing in all three species. Of the regions that were present, *a1* was significantly truncated in all three unisexual species, with an in-frame stop codon in the position that would have been the C residue of the CpaX domain (Fig. 4, Figs S2, S3 and S4). The remainder of the gene region was similar to that from the heterothallic species and had the stop codon not been present, the encoded protein would likely have been functional as an a-factor pheromone.

**Fig. 6:**
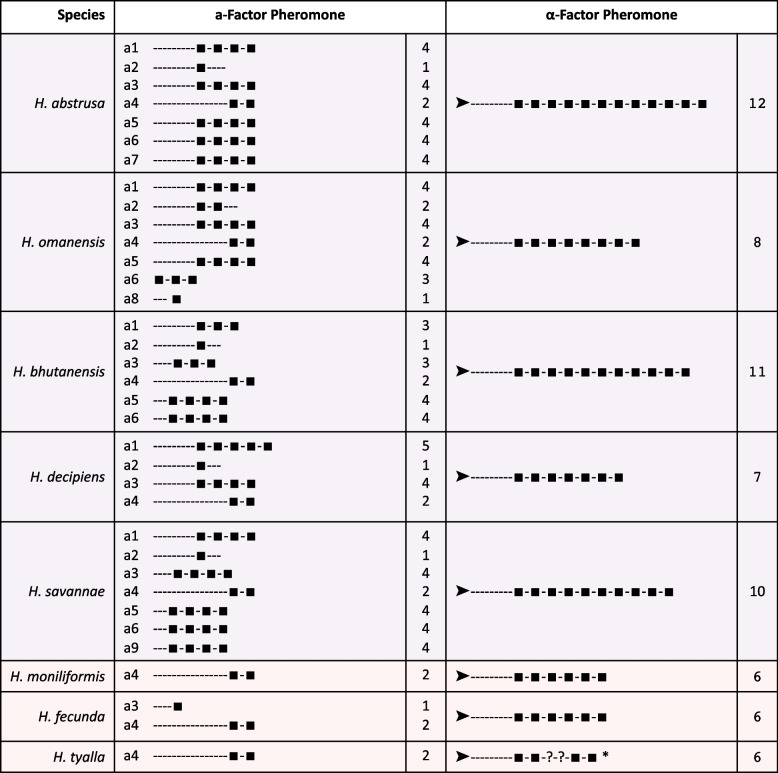
The structure of the *Huntiella* a- and α-factor pheromone pre-proteins. Each block in both the a- and α-factor pheromone columns represents a putative mature repeat, while the number of repeats present in each pheromone is indicated in the next column. The arrows in the α-factor pheromone column represent the signal peptide identified in these proteins. While the structure schematics are not drawn to scale, they are representative of the length of the non-repeat harboring N-terminal of the **a**-factor pheromone. * The *H. tyalla* α-factor pheromone contig could not be fully assembled. The sequences of each putative repeat are included in Figs. S2 and S9

The region that harbored the *a3* gene differed significantly between the heterothallic and unisexual species (Fig. 4 and Fig. S5). While the region typically harbored a gene encoding a protein with up to four copies of the mature pheromone peptide in the heterothallic species, *H. fecunda* was the only unisexual species that harbored an intact *a3* gene and it encoded a protein with only a single copy of the mature pheromone peptide (Fig. 6, Fig. S2). Furthermore, the *H. moniliformis* and *H. tyalla a3* region harbored a mutated version of the gene, with a mutation present within the start codon, making it unlikely that this sequence would be recognised by the ribosome for translation. The *a4* gene was the only a-factor pheromone gene that encoded a full-length protein in all three unisexual species (Figs. 4 and 5). The gene encoded a protein with two recognizable copies of the mature pheromone peptide.

#### The second *a*-factor pheromone locus

Additional putative a-factor pheromone genes, named *a5* to *a9,* were present at different genomic locations in some of the heterothallic *Huntiella* species studied (Fig. 5). While the flanking genes of these additional pheromones were fairly well-conserved, there have been numerous rearrangements at this locus. The a-factor pheromones have thus been named based on their presence/absence, order within the *H. abstrusa* locus, and subsequent homology.

In four of the five heterothallic species considered here, *a5* was associated with genes encoding a putative alkaline phosphatase family protein (AP) and an unknown protein (U1) at its 3’ end. Significant rearrangements had occurred amongst the flanking genes present at the 5’ end of the *a5* pheromone, with up to six genes being inverted in the *H. abstrusa* and *H. omanensis* loci compared to the remaining heterothallic species (Fig. 5). Notably, the *a5* homolog was lost from this locus once in the heterothallic *H. decipiens* and once in the ancestor of the three unisexual species. Given the position of the locus within fully assembled contigs, it is unlikely to have been an assembly error and thus most likely represents a true gene deletion within these species. Due to the complete absence of this gene from these genomes and the fact that there were no genetic signals left at this locus, it was not clear how it was lost.

The *a6* homolog was flanked by a laccase-1-encoding gene (Lac) at its 3’ end in the eight *Huntiella* species considered here (Fig. 5). Despite this conservation, the *a6* genes present in *H. decipiens* and the three unisexual species were truncated. In *H. decipiens*, the truncating stop codon occurred within the region encoding the first of the predicted mature pheromone peptides. In contrast, the stop codons in the *H. moniliforms* (Fig. S4), *H. fecunda,* and *H. tyalla a6* genes were at a position comparable to the premature stop codon in the *a1* gene of these species.

The *a7, a8* and *a9* homologs were each only present in a single species, *H. abstrusa*, *H. omanensis*, and *H. savannae*, respectively (Fig. 5). The *H. abstrusa a7* gene was flanked by genes encoding a urease accessory protein (UA) and a heterokaryon incompatibility protein (HI). Both the *H. omanensis a8* and *H. savannae a9* genes were present upstream of the *a6* gene. The *a6* and *a8* genes from *H. omanensis* are quite different from the **a**-factor genes in the other species and potentially represent a recent duplication or translocation. The *a8* gene encodes a short protein that is very similar to the N-terminal of the *H. abstrusa* a6 protein, while the *H. omanensis a6* gene encodes a protein that is comparable the C-terminal of the *H. abstrusa* a6 protein (Fig. 5).

There was significant intra- and inter-species similarity amongst the *a5* to *a9* genes, and particularly amongst the proteins that these genes encode. In intra-species comparisons, the *a5*, *a6* and *a7/a8* genes from *H. abstrusa* and *H. savannae*, and the *a5* and *a6* genes from *H. bhutanensis*, showed percent identities of >96%. Furthermore, the proteins encoded by these genes showed percent identities of 100% in intra-species comparisons, suggesting that these are very recent duplications and indicating that strong selection pressures have ensured that the protein sequences remain conserved.

### Structure of the *Huntiella* a-factor pheromone proteins

The structure of many of the *Huntiella* a-factor pheromone proteins was similar to the a-factor pheromone in the *Fusarium fujikuroi* species complex [23] and the h-type pheromone in various *Trichoderma* species [24]. This was due to their harboring multiple CpaX domains, each preceded by a highly conserved peptide that was thought to represent the mature pheromone (Figs. 1 and 6, Fig. S2). The pheromone with the most mature repeats was *a1* in *H. decipiens*, which harbored five mature repeats, while the *a2* pheromone from all of the heterothallic species (except *H. omanensis*) harbored only a single repeat.

The putative pheromone repeats were highly conserved within species. While the CpaX domains varied from repeat to repeat, the peptide preceding this conserved domain often differed by only a few residues. This peptide always began with an arginine residue, almost always ended in a proline residue, and always had a glutamine residue at position five (Fig. S2). Additionally, some repeats were shared by multiple species. For example, RSINQSSP was present in pheromones of *H. abstrusa* and *H. decipiens*, RGVDQSNP was present in pheromones of *H. omanensis* and *H. bhutanensis*, and RGVTQAPP was present in the *H. moniliformis*, *H. fecunda*, and *H. tyalla* pheromones.

### Expression of the multiple a-factor pheromone genes

In order to determine whether the multiple a-factor pheromone genes were expressed, various read mappings were generated using RNAseq data from two heterothallic species, *H. abstrusa* and *H. omanensis,* and one unisexual species, *H. moniliformis*. This analysis was challenging given the sequence similarity between the various homologs. To overcome this, mapping stringencies of 1.0 for both length and similarity fractions were needed to produce usable results for both heterothallic species. For similarity fractions of 0.9 and lower, an inordinately large number of reads were assigned to the incorrect homolog. The same was not true for the unisexual species, *H. moniliformis*, for which usable mappings could be generated with mapping stringencies of 1.0 and 0.9 for length and similarity fraction, respectively. This is likely due to the presence of fewer copies of this gene as well as fewer repeats per gene.

RNA reads mapped to many of the seven a-factor pheromone genes in both *H. abstrusa* and *H. omanensis*, indicating that they are expressed (Figs. S6 and S7). However, there appeared to be differences in the expression levels of each gene. For example, the *H. abstrusa a3* and *a6* genes were more highly expressed than *a1* and *a2*. Similarly, the *H. omanensis a1* and *a5* genes were expressed at higher levels than *a2* and *a3*. For both heterothallic species, *a4* was expressed at very low or undetectable levels.

It has previously been reported [22] that *a4* was expressed in *H. moniliformis* (Fig. S8). In the present study, there was also evidence for the expression of *a1* and *a6*, despite the fact that these transcripts would likely not be translated into functional proteins. No reads mapped to *a3*, strongly indicating that this gene is not transcribed in *H. moniliformis* or that it is expressed at undetectable levels.

### Unisexual *Huntiella* species have fewer mature α-factor peptide repeats

The genes encoding the α-factor pheromone in *H. omanensis*, *H. bhutanensis,* and *H. moniliformis* were identified in a previous study [21]. In the present study, the **α**-factor homologs were identified from the remaining five species. However, the full-length gene could not be extracted from the *H. tyalla* genome because the contig harboring the homolog was not fully assembled.

The structure of the *Huntiella* α-factor pheromone was comparable to that in most other ascomycete fungi and harbored a signal peptide at the N-terminal, various KEX1/2 processing sites (RR) and repeats of the mature pheromone peptide (Figs. 1 and 6, Fig. S9). The repeat units were predicted to be 11 aa in length and were well-conserved across the genus. This was true within the heterothallic *Huntiella* spp., where NSNGGLPGELL and DSNGGLPGELL were common, and within the unisexual fungi, where DANGGLPGELF and DAWGGLPGELF were present. Notably, the heterothallic species had more repeats per protein than the unisexual species. These species had an average of 9.6 repeats, with a minimum of seven repeats (*H. decipiens*) and a maximum of 12 repeats (*H. abstrusa*). In contrast, all three unisexual *Huntiella* species only had six repeats each.

## Discussion

A combination of whole genome and transcriptome sequencing was used to identify genetic elements that differ between heterothallic and unisexual *Huntiella* species. Previous studies have shown that the unisexual *H. moniliformis* harbors a truncation in the *MAT1-2-7* gene that was not shared by heterothallic *Huntiella* species [21, 29]. It had also been shown that the protein sequence representing the putative *H. moniliformis* a-factor pheromone had a different structure to those representing the same protein from *H. omanensis* and *H. bhutanensis* [21]. Results of this study showed that the *MAT1-2-7* truncation is present in two additional unisexual species, *H. fecunda* and *H. tyalla,* and that it is absent in a wider range of heterothallic *Huntiella* species than had previously been considered. Furthermore, the structural differences between the a-factor pheromones were shown to be the result of multiple copies of the pheromone being present in the genomes of these species.

The two primary *MAT* genes are essential for sexual development in many fungi, including those in the Sordariomycetes [as reviewed by [18]], the Leotiomycetes [37, 38] and the Dothideomycetes [39]. In contrast, the secondary *MAT* genes typically show a pattern of dispensability [21]. Interestingly, there is evidence to suggest that *MAT* genes, specifically the secondary *MAT* genes, are under less stringent selection pressure in homothallic species compared to related heterothallic species [40]. For example, while all five *MAT* genes are retained in the heterothallic *N. crassa*, *MAT1-1-3* is missing in the homothallic *Neurospora terricola* and disrupted in the unisexual *Neurospora lineolata* and *N. africana*, and *MAT1-1-2* is disrupted in the unisexual *Neurospora dodgei* [21, 40]. It is thus not surprising that a full-length and presumably functional *MAT1-2-7* is present in the three heterothallic *Huntiella* species for which a MAT1-2 genome is available, while being significantly truncated in the three unisexual species. *MAT1-2-7* has been experimentally disrupted in *H. omanensis* and was shown to be essential for sexual reproduction [41, 42], however it is unclear what role it plays in the sexual cycle. Further research considering the molecular function and cellular localization of this protein should be conducted to elucidate why the gene is dispensable in homothallic fungi while appearing to be essential in a variety of heterothallic species.

The discovery of multiple genes encoding for putative a-factor pheromones in various heterothallic and unisexual *Huntiella* species was surprising because such massive gene duplication is not known in other filamentous species. This is a well-known phenomenon in the Saccharomycetes and Taphrinomycetes, but even then, the a-factor pheromone is encoded by three or fewer genes [23]. In most cases, these genes encode slightly different proteins, although the mature pheromone peptide is highly conserved. To the best of our knowledge, the only other known example of multiple copies of the a-factor pheromone in the Pezizomycotina is in *Cryphonectria parasitica* [43] which harbors two identical copies of this gene. However, many of the Pezizomycotina mating pheromones were identified before genome sequencing became common place. It is thus likely that similar pheromone duplications have occurred in other species but that they have simply not been formally described.

One explanation for the presence of multiple copies of the a-factor pheromone gene is that it allows for the production of suitable quantities of the mature pheromone factor [44]. A similar rationale may explain the repetitive nature of the ascomycete α-factor pheromone, which ensures that the expression of a single gene can result in the production of sufficient quantities of mating pheromone. However, unlike many other ascomycetous a-factor proteins, the *Huntiella* **a**-factor pheromone is also repetitive in nature. Thus, the fact that *Huntiella* species harbor multiple copies of this repetitive gene is surprising, and the implication is that these fungi are capable of secreting large amounts of a-factor pheromone. A possible explanation is that this pheromone has been co-opted for other, non-mating processes, such as quorum sensing as has been documented in *Fusarium oxysporum* [45]. However, given that this phenomenon seems to be particularly true of the heterothallic fungi and that this gene is expressed exclusively by MAT1-2 isolates [22], it is likely that the pheromone is primarily important for sexual reproduction.

An alternative explanation for the duplication and repetitive nature of the pheromone genes could lie in the ecology of these fungi as they must grow and produce sexual structures rapidly in order to accommodate their reliance on insect vectors. Fungi in the Ceratocystidaceae, which accommodates *Huntiella*, produce long necked ascomata that exude sticky drops of ascospores that easily attach to insects and thus facilitate dispersal to new substrates [25, 46]. The production of huge amounts of pheromone may act to stimulate sexual development on a much shorter time scale, thereby ensuring that the structures required for insect interaction can be produced and ensuring the continuation of the life cycle. While this hypothesis is speculative and requires experimental testing, the possibility that pheromone production could influence culture fertility and the rapid production of spores for dispersal would substantially improve our understanding of the biology of the Ceratocystidaceae.

The mechanisms that underlie unisexual reproduction in filamentous ascomycete fungi are not fully understood. Based on differences between the heterothallic *H. omanensis* and the unisexual *H. moniliformis*, it was previously suggested that *MAT1-2-7* truncation and/or mating type-independent pheromone expression was responsible for unisexual reproduction [22, 29]. The present study identified other notable differences between heterothallic and unisexual species of *Huntiella*. These included the number and structure of a-factor pheromone-encoding genes and the number of repeats in the α-factor pheromone.

The observed degeneration of pheromones in unisexual *Huntiella* species may be the result of changing selection pressures on the genes responsible for pheromone production. The primary function of the pheromone pathway is to facilitate mating partner interactions, and producing these highly processed peptides is energy-intensive [47, 48]. Previous studies have shown that pheromone expression is subject to sexual selection, because individuals that produce higher quantities of expensive pheromones are more attractive to potential mating partners [48]. Therefore, the evolution of self-fertility in these fungi would likely have led to a relaxation in the selection pressures responsible for excess pheromone production, resulting in a decrease in pheromone expression capabilities. This decrease could have been facilitated by the degeneration or deletion of genes encoding the two mating pheromones, the result of which has been illustrated in this study.

**Fig. 7:**
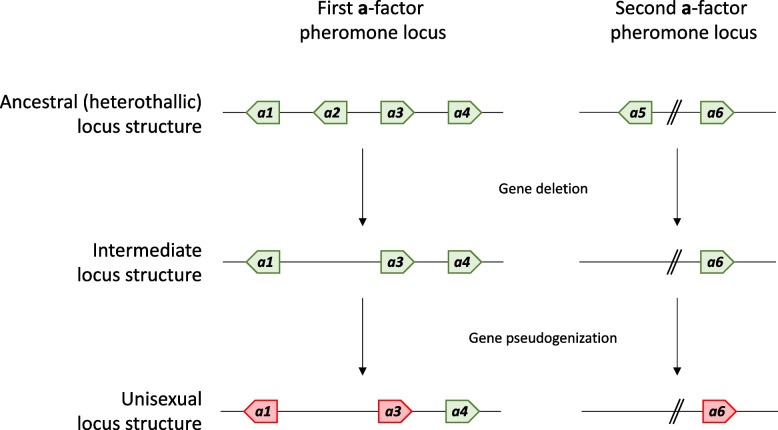
The predicted model for the loss of the a-factor pheromone at both the first and second loci in the unisexual *Huntiella* species. At both loci, there have been instances of gene deletion (*a2* and *a5*) as well as gene pseudogenization (*a1*, *a3*, and *a6*), both of which have resulted in fewer copies of the a-factor pheromone in the three unisexual species compared to the heterothallic species

We have hypothesized that unisexual species of *Huntiella* have undergone gene loss rather than the heterothallic species having undergone gene gain. The diverse heterothallic species included in the present study had very similar a-factor pheromone loci, suggesting that this structure represents the ancestral state of these genomic regions (Fig. 7). From there, it appears that successive events of gene loss of the a-factor pheromone have taken place in the unisexual species. The *a2* and *a5* genes have been deleted from the first and second loci, respectively, while the *a1*, *a3*, and *a6* genes appear to be undergoing pseudogenization via the introduction of premature stop codons and disrupted start codons. However, the three unisexual species also lacked *a7*, *a8* and *a9* homologs that cannot be linked to specific gene loss events given their inconsistent presence among the heterothallic species. It is thus possible that the presence of these three genes is the result of gene gain in specific heterothallic species; likely via duplication and/or translocation events. Given the repetitive nature of these genes, phylogenetic analyses were challenging, and it was not possible to identify the origin of these genes and which homologs resulted from recent duplications.

The heterothallic *H. decipiens* represents an interesting deviation from the other *Huntiella* species as it has pheromone locus characteristics in common with both the heterothallic and unisexual species considered here. Similar to the other heterothallic species, *H. decipiens* possessed all four **a**-factor pheromone genes at the first locus. However, like the three unisexual species, homologs of the *a5* and *a6* genes were missing and truncated, respectively. Unfortunately, other than the taxonomic description of the species [49] and subsequent genome announcement [33], no research has been conducted on *H. decipiens.* It is also worth noting that the genome sequence available for *H. decipiens* represents a MAT1-1 isolate and that unisexual MAT1-1 isolates have not been described for any of the *Huntiella* species. Thus, while it is possible that the observed differences at the pheromone loci mean that this heterothallic species may also be capable of unisexual reproduction, further research considering the mating biology of this fungus and the structure of the *MAT* locus in MAT1-2 individuals is required.

It is difficult to ascertain whether the disruption of the *MAT1-2-7* gene, the mating type-independent pheromone expression, and/or the gene/repeat loss associated with the pheromones were the mechanisms that gave rise to the transition from heterothallism to unisexuality or whether they were a consequence of this transition. While many of the genetic pathways that govern sexual reproduction are conserved between heterothallic and homothallic species, the initial stages of sexual development, including partner recognition, are necessarily different. It seems likely that the significant change from self-sterility to self-fertility would be governed by a change to genes that are involved in this initial interaction and as such, alteration to the mating pheromones may very likely be involved. However, it is also possible that other, unknown genetic mechanisms have led to the transition. Once self-fertile, there would then have been a reduced need for the pheromone response pathway, and it could thus evolve independently of its original function. Further research, including functional characterization of these genes, is needed to fully understand the mechanisms of transition between sexual strategies in this group of fungi and others.

Transitions between homothallism and heterothallism may also have been influenced by other components of the pheromone response pathway. Although the pheromone receptors do not appear to differ significantly between heterothallic and unisexual *Huntiella* species [21], distinctions may exist in the G-protein subunits and kinases that are activated in response to the pheromone-receptor binding. In unisexual species, the pheromone response pathway could have been repurposed for an entirely different function, as they do not need to attract mating partners. Consequently, there may be noticeable dissimilarities in the gene sequences and expression profiles of other pathway components.

A limitation of this study is that unisexuality has ostensibly evolved only once in *Huntiella*, because the unisexual species form a monophyletic clade. Additionally, the similarity of the a-factor pheromone loci amongst the unisexual species suggests that much of the degeneration of these genes occurred only once, in the ancestor of these unisexual fungi. While this fact does not alter the results or their interpretation, it may limit the generalization of these results to other instances of sexual strategy transition because it represents only a single transition from heterothallism to homothallism.

## Conclusions

Transitions between different sexual strategies are common throughout the ascomycete fungi [9]. While there is some debate regarding the topic, it seems likely that the overall pattern of evolution has been from heterothallism towards homothallism. Given that many of the physiological and morphological changes associated with sexual development are often shared between heterothallic and homothallic fungi, it is likely that genes and proteins involved in the initial phases of sexual reproduction would be involved in a change of sexual strategy [18]. In this study, we have provided additional evidence that the transition from heterothallism to homothallism was associated with changes in the *MAT1-2-7* gene as well as both pheromone genes. The inclusion of a diverse set of heterothallic fungi and two additional unisexual species supports previous hypotheses proposed at a time when data were available for only three species.

## Methods and materials

### Isolates used

A total of three isolates were used in this study. Of these, two were used for genome sequencing. The first was an isolate of *H. fecunda* (CMW 49301), a species that was recently described as unisexual as part of a greater cataloguing of *Huntiella* species in southern China [28]. The second was an isolate of *H. tyalla* (CMW 28920), a species for which no sexual strategy had been documented. The third isolate was used for transcriptome sequencing and was *H. abstrusa* (CMW 21092), a species that has been described as heterothallic [27]. Cultures of all three isolates were maintained at room temperature on 2% (w/v) malt extract agar supplemented with thiamine hydrochloride (100 mg.L-1) and streptomycin sulphate salt (150 mg.L-1) for the duration of the study. These cultures are preserved at the culture collection (CMW) of the Forestry and Agricultural Biotechnology Institute (FABI), University of Pretoria, South Africa.

The sexual strategy of *H. tyalla* was determined by generating single hyphal tip isolates and incubating these under conditions conducive to sexual reproduction. This included maintaining them at room temperature, in unsealed Petri dishes within plastic boxes containing silica sand crystals that were used to decrease the relative humidity. These cultures were maintained as above for up to two weeks, during which they were visually inspected for the production of ascomata. The sexual strategy of *H. fecunda* was confirmed using the same method.

### Data used

A total of eight genomes were used for the analyses described here (Table 1). The genomes of *H. abstrusa, H. omanensis*, *H. bhutanensis*, *H. decipiens*, *H. savannae,* and *H. moniliformis* were available prior to this study. The genomes of *H. fecunda* and *H. tyalla* were sequenced for the present study and have been deposited into the Genome database of the National Centre for Biotechnology Information (NCBI).

**Table 1:** Isolates and data used in this study

Species	Isolate	Mating Type	Data Type	Accession Number	Reference
*H. abstrusa*	CMW 21092	MAT1-2	DNA	JAJNMT000000000.1	[34]
			RNA	PRJNA894346	*This study*
*H. omanensis*	CMW 11056	Mixed	DNA	JSUI00000000.1	[30]
			RNA	PRJNA385659	[22]
*H. bhutanensis*	CMW 8217	Mixed	DNA	MJMS00000000.1	[32]
*H. decipiens*	CMW 30755	MAT1-1	DNA	NETU00000000.1	[33]
*H. savannae*	CMW 17300	MAT1-1	DNA	LCZG00000000.1	[35]
*H. moniliformis*	CMW 10134	MAT1-2	DNA	JMSH00000000.1	[31]
	CMW 36919	MAT1-2	RNA	PRJNA385659	[22]
*H. fecunda*	CMW 49301	MAT1-2	DNA	JAPHQJ000000000.1	*This study*
*H. tyalla*	CMW 28920	MAT1-2	DNA	JAPHQI000000000.1	*This study*

Additionally, numerous RNAseq datasets were also used as detailed below (Table 1). The datasets for *H. omanensis* and *H. moniliformis* were available prior to this study [22] while the *H. abstrusa* dataset was produced for the present study and has been deposited into the sequence read archive (SRA) database of the NCBI.

### Genomic DNA extraction and sequencing

Cultures of *H. fecunda* and *H. tyalla* were grown in 2% malt extract broth at 25°C with shaking at 120 rpm for up to five days. Mycelium was collected by centrifugation at 4 000 rpm for 10 mins. DNA was extracted from the collected mycelium using a rapid salt-extraction protocol with modification [50, 51]. The quantity and quality of the extracted DNA was assessed using a Qubit and via electrophoresis using a 1% (w/v) agarose gel, respectively. The extracted gDNA was used to construct Illumina libraries using the TruSeq PCR free library kit (550 bp median insert size) and was sequenced at Macrogen (Seoul, Korea) on the HiSeq 2500 platform.

### Genome assembly

Whole genome sequencing yielded just over 20 million raw reads from *H. fecunda* and almost 28 million raw reads from *H. tyalla* (Table S2). These reads were subjected to quality analysis using FastQC v0.11.5. Poor quality data were removed using Trimmomatic v0.39 and the trimmed data were subjected to a second round of quality analysis using FastQC v0.11.5. A total of 19.6 million reads (96.28%) remained from *H. fecunda* while 27.1 million reads (97.67%) remained from *H. tyalla* (Table S2).

High quality reads were subsequently assembled using SPAdes v3.13.0 [36]. Genome completeness was assessed using BUSCO v4.0.6 [52]. The *fungi_odb10*, *ascomycota_odb10*, and *sordariomycetes_odb10* lineage datasets were used in three separate BUSCO runs. Genome assembly statistics were generated using QUAST v5.1 [53] and gene annotations were generated using AUGUSTUS v3.2.3 and the *Fusarium graminearum* gene models [54]. All of the scripts and parameters used for the genome assembly are available in Supplementary file S1.

In addition to the genome assemblies produced in this study, the raw data from *H. fecunda* were also used to construct the second a-factor pheromone locus. This region had not been assembled in full using SPAdes. The trimmed raw data were mapped to the homologous locus from *H. moniliformis* using the *Map reads to contigs* option in the *De Novo Sequencing* module in CLC Genomics V22, with default settings. The reads that mapped to this locus were extracted and used to assemble the *H. fecunda* locus using the *De novo assembly* option in the same CLC Genomics module*,* with default settings.

### Phylogenetic analyses

A phylogeny was generated using the three gene regions typically used to differentiate species in *Huntiella*: β-tubulin (BT1), internal transcribed spacer (ITS), and translation elongation factor (TEF1). These regions were extracted from each of the genomes used in this study and combined with homologous sequences from an additional 29 *Huntiella* species [26]. These sequences were independently aligned in MAFFT v7.0, using default settings and the alignments were manually curated.

Model testing for each of the alignments was conducted in MrModelTest2 V2.4, after which the three alignments were concatenated into a single dataset. MrBayes V3.2.7 was subsequently used to conduct Bayesian inference (BI) analyses [55]. These analyses were run for one million generations, with ten parallel runs and four chains. Trees were sampled every 100 generations, with 25% of the sampled trees being discarded as burn-in. Posterior probabilities were calculated from the remaining trees. The final tree was visualized in FigTree v1.4.4 [56]. The gene alignments and all parameters for this analysis can be found in Supplementary file S2.

### Gene identification and comparisons

Two sets of mating-related genes were identified and compared for this study; the mating-type (*MAT*) genes and the two pheromone genes. These genes had previously been identified in *H. omanensis*, *H. bhutanensis*, and *H. moniliformis* [21] and could thus be used as BLASTn and tBLASTn queries against the remaining five genomes. BLAST searches were conducted in CLC Main Workbench V22.

### RNA extraction and sequencing

RNA was extracted from sexually reproducing cultures of *H. abstrusa.* These cultures were induced to sexually reproduce as described above. A mixture of mycelium, proto-ascomata and ascomata was harvested from nine cultures- representing three biological and three technical replicates. This tissue was flash frozen with liquid nitrogen and total RNA was extracted as previously described [22]. The integrity, quality and concentration of the RNA was assessed and the mRNA-enrichment and library preparation were conducted as previously described [22]. RNA sequencing was performed at the Central Analytical Facilities (CAF) at Stellenbosch University.

### Gene expression analyses

The RNAseq datasets from *H. abstrusa*, *H. omanensis,* and *H. moniliformis* were used to assess the expression of the various genes identified. Additionally, these data were used to support the predicted gene models and to confirm the presence of deletions and mutations, particularly those that resulted in functional changes to the encoded protein, eg: abolished start codons and premature stop codons.

All RNAseq analyses were performed in CLC Genomics Workbench V22 using the *Prepare Sequencing Data* and *De Novo Sequencing* modules. The raw RNA reads were trimmed using default settings and mapped to the contigs harboring the genes of interest. The default mapping settings were used for the *MAT* locus contigs, except for the length and similarity fractions, which were both set to 0.9. These stringent settings were suitable for this analysis because the *MAT1-2-7* gene does not possess introns, which would otherwise require a less stringent length fraction parameter to ensure that reads spanning the intron would be mapped.

Analysis of the a-factor pheromone was challenging due to the fact that multiple copies of the gene were identified in each genome. The high sequence similarity made it difficult to assign reads to one homolog over another. In order to best deal with this problem, numerous mapping analyses were conducted, each with varying levels of parameter stringency. The length fraction was maintained at 1.0 given the absence of introns in these genes, while the similarity fraction was set at 0.8, 0.9 and 1.0. This was particularly important for *H. moniliformis* because isolate CMW 10134 was used to generate the genome sequence and isolate CMW 36919 was used to generate the transcriptomic data. Consequently, single nucleotide polymorphisms were likely to be present in these two datasets.

## Supplementary Information

**Additional file 1:** **Fig. S1.** RNA mapping to confirm the single nucleotide deletion in the* H. moniliformis MAT1-2-7*. **Fig. S2. **The structure of the *Huntiella* a-factor pheromone proteins and the sequence of the putative mature repeats. **Fig. S3.** An alignment of the *a1* a-factor pheromone factor genes from all eight *Huntiella* species considered in this study. **Fig. S4.** RNA mapping to confirm in frame stop codons in the *a1* and *a6* a-factor pheromone factor genes from the unisexual *H. moniliformis*. **Fig. S5.** An alignment of the *a3* a-factor pheromone from all eight *Huntiella* species considered in this study. **Fig. S6.** RNA mapping to determine expression of the multiple a-factor pheromone genes from *H. abstrusa*. **Fig. S7.** RNA mapping to determine expression of the multiple a-factor pheromone genes from *H. omanensis*. **Fig. S8.** RNA mapping to determine expression of the multiple a-factor pheromone genes from *H. moniliformis*. **Fig. S9. **The structure of the *Huntiella* α-factor pheromones from the various *Huntiella* species along with the sequences of the putative mature repeats.

**Additional file 2:** **Supplementary File 1. **All of the scripts and parameters used for the genome assemblies produced in this study.

**Additional file 3:** **Supplementary File 2. **The gene alignments and all parameters for the phylogenetic analysis conducted in this study.

**Additional file 4:** **Table S1.** Genome sequencing and assembly statistics. **Table S2.** Comparisons of *Huntiella* genome statistics. **Table S3.** Gene present at the second a-factor pheromone locus.

## Data Availability

All data used and generated in this study are available either in the Genome or Sequence Read Archive (SRA) databases of the NCBI or in the Supplementary Data associated with this manuscript. The accession numbers of the genomes are as follows: *H. abstrusa* (JAJNMT000000000.1), *H. omanensis* (JSUI00000000.1), *H. bhutanensis* (MJMS00000000.1), *H. decipiens* (NETU00000000.1), *H. savannae* (LCZG00000000.1), *H. moniliformis* (JMSH00000000.1), *H. fecunda* (JAPHQJ000000000.1) and *H. tyalla* (JAPHQI000000000.1). The BioProject numbers of the RNAseq raw reads are as follows: *H. abstrusa* (PRJNA894346), *H. omanensis* (PRJNA385659) and *H. moniliformis* (PRJNA385659).
