# Supplementary material for: Structure and number of mating pheromone genes is closely linked to sexual reproductive strategy in Huntiella
Source: BMC Genomics. 2023 May 13;24:261. doi: 10.1186/s12864-023-09355-9 (PMC10182648; doi:10.1186/s12864-023-09355-9)
Supplement: Supplementary file 1 — Additional file 1: Fig. S1. RNA mapping to confirm the single nucleotide deletion in the H. moniliformis MAT1-2-7. Fig. S2. The structure of the Huntiella a-factor pheromone proteins and the sequence of the putative mature repeats. Fig. S3. An alignment of the a1 a-factor pheromone factor genes from all eight Huntiella species considered in this study. Fig. S4. RNA mapping to confirm in frame stop codons in the a1 and a6 a-factor pheromone factor genes from the unisexual H. moniliformis. Fig. S5. An alignment of the a3 a-factor pheromone from all eight Huntiella species considered in this study. Fig. S6. RNA mapping to determine expression of the multiple a-factor pheromone genes from H. abstrusa. Fig. S7. RNA mapping to determine expression of the multiple a-factor pheromone genes from H. omanensis. Fig. S8. RNA mapping to determine expression of the multiple a-factor pheromone genes from H. moniliformis. Fig. S9. The structure of the Huntiella α-factor pheromones from the various Huntiella species along with the sequences of the putative mature repeats. [file 12864_2023_9355_MOESM1_ESM.pdf]

FIGURE S1

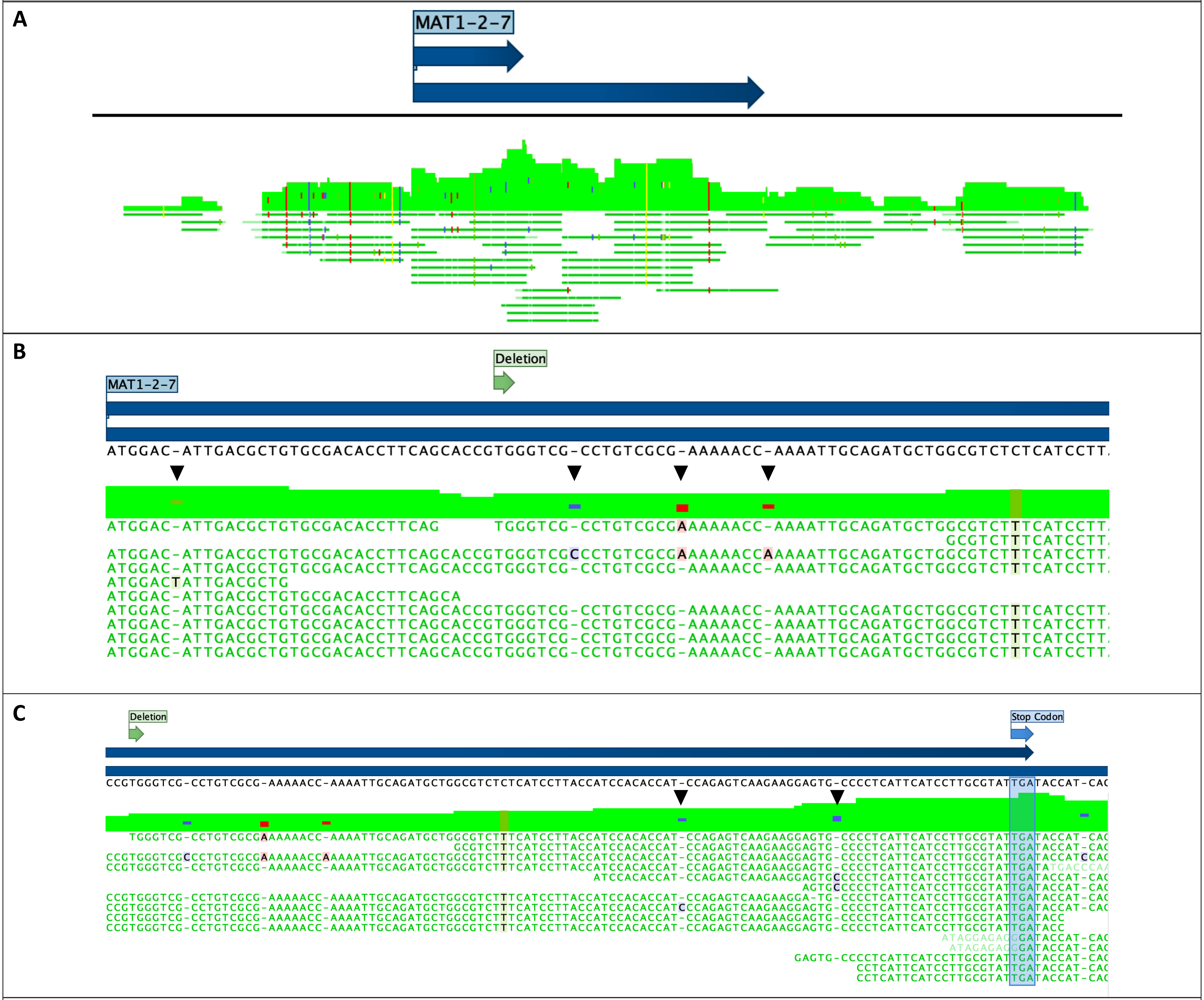

Fig S1: RNA mapping to confirm the single nucleotide deletion in the *H. moniliformis MAT1-2-7*. This mapping was generated using three sets of RNAseq data from a sporulating *H. moniliformis* isolate (Wilson et al 2018). A) Overview of the mapping across the entire gene. B) Mapping of the 5' region of the gene, indicating the start codon as well as the region where the deletion took place (indicated by the light green annotation). C) Mapping of the 3' region of the gene, indicating the region where the deletion took place as well as the in frame stop codon (indicated by the light blue annotation and box). There are various SNPs apparent when comparing the RNAseq reads to the assembled contig. Some of these (particularly those indicated with a black triangles) are likely sequencing errors produced during the transcriptome sequencing- especially considering they often occur in homopolymeric regions. These errors are often only supported by one or two reads. The mapping was produced using the CLC Genomics Workbench V22.0 Map Reads to Contigs function within the De Novo Sequencing module. The parameters were all set to default, except the minimum length and similarity fractions, which were each set to 0.9.

FIGURE S2

Species	Protein structure
H. abstrusa	a1 ---------RGVDQSSG**CSVM** RGVDQSSG**CSVM** RGVDQSSG**CTLM** RGVDQSSG**CTLM**<br>a2 ---------RGVDQSSG**CSVM**---<br>a3 ---------RGVDQSSG**CSVM** RGVDQSSG**CSVM** RGVDQSSG**CTVM** RGVDQSSG**CTVM**<br>a4 ----------------RSINQSSP**CTVM** RSINQSSP**CNVM**<br>a5 ---------RTVNQSGP**CNVM** RTVNQSGP**CNVM** RTVNQSGP**CNVM** RTVNQSGP**CNVM**<br>a6 ---------RTVNQSGP**CNVM** RTVNQSGP**CNVM** RTVNQSGP**CNVM** RTVNQSGP**CNVM**<br>a7 ---------RTVNQSGP**CNVM** RTVNQSGP**CNVM** RTVNQSGP**CNVM** RTVNQSGP**CNVM**
H. omanensis	a1 ---------RGVDQSNP**CAVM** RGVDQSNP**CAVM** RGVDQSNP**CTVM** RGVDQSNP**CTLM**<br>a2 ---------RGVDQSNG**CAVM** RGVDQSNG**CTVM**--<br>a3 ---------RGVDQSNP**CAVM** RGVDQSNP**CAVM** RGVDQSNP**CAVM** RGVDQSNP**CTVM**<br>a4 ----------------RTVNQSSP**CTVM** RSINQSTP**CNVM**<br>a5 ---------RVANQSNP**CNVM** RTANQSNPCTVM RTGNQSNPCVVM RTGNQSNPCNVM<br>a6 MRTGNQSNPCTVM RTGNQSNPCVVM RTGNQSNPCNVM<br>a7 --- RVANQSNP**CNVM**
H. bhutanensis	a1 ---------RGVDQSNP**CNVM** RGVDQSNP**CAVM** RGVDQSNP**CTVM**<br>a2 ---------RGVDQSNP**CNVM**--<br>a3 ----RGVDQSNP**CSVM** RGVDQSNP**CAVM** RGVDQSNP**CTVM**<br>a4 ----------------RTFNQSSP**CTVM** RTINQSSP**CNVM**<br>a5 ---RAASQSNP**CNVM** RAASQSNP**CTVM** RAASQSNP**CTVM** RAASQSNP**CNVM**<br>a6 ---RAASQSNP**CNVM** RAASQSNP**CTVM** RAASQSNP**CTVM** RAASQSNP**CNVM**
H. decipiens	a1 ---------RGVDQSNG**CSVM** RGVDQSNG**CTVM** RGVDQSNG**CTVM** RGVNQSNG**CTVM**<br>RGVNQSNG**CTLM**<br>a2 ---------RGVDQSNG**CAVM**--<br>a3 ---------RGVDQSNG**CSVM** RGVDQSNG**CTVM** RGVDQSNG**CTVM** RGVNQSNG**CTLM**<br>a4 ----------------RTVNQSGP**CTVM** RSINQSSP**CNVM**
H. savannae	a1 ---------RGVDQSTP**CSVM** RGVDQSTP**CNVM** RGVDQSTP**CNVM** RGVDQSTP**CTVM**<br>a2 ---------RGVDQSTP**CSVM**--<br>a3 ----RGVDQSTP**CSVM** RGVDQSTP**CNVM** RGVDQSTP**CNVM** RGVDQSTP**CNVM**<br>a4 ----------------RSVNQSTP**CTVM** RSINQSTP**CNVM**<br>a5 ---RGVNQSTP**CNVM** RGVNQSTP**CNVM** RGVNQSTP**CNVM** RGVNQSTP**CNVM**<br>a6 ---RGVNQSTP**CNVM** RGVNQSTP**CNVM** RGVNQSTP**CNVM** RGVNQSTP**CNVM**<br>a7 ---RGVNQSTP**CNVM** RGVNQSTP**CNVM** RGVNQSTP**CNVM** RGVNQSTP**CNVM**
H. moniliformis	a4 ----------------RGVTQAPP**CNVM** RGVTQAPP**CNVM**
H. fecunda	a3 ----RGVTQAPP**CNVM**<br>a4 ----------------RGVTQAPP**CNVM** RGVTQAPP**CNVM**
H. tyalla	a4 ----------------RGVTQAPP**CNVM** RGVTQAPP**CNVM**

Fig S2: The structure of the *Huntiella* a-factor pheromone proteins and the sequence of the putative mature repeats. The repeat units are represented by coloured squares and while the structure schematics are not drawn to scale, they are representative of the length of the non-repeat harbouring N-terminal.

FIGURE S3

```
                                   20                    40                    60                    80
                                    |                     |                     |                     |
H. abstrusa     ATGGCCGCTA TCAAGAACAC CACCTCCTCC AAGAACGCCG CCCGCGGCGT CGACCAATCC AGCGGATGCA GCGTCATGCG 80
H. omanensis    ATGGCCGCTA TCAAGAACAC CACCACCTCC AAGAACGCCG CCCGCGGCGT TGACCAGTCC AACCCCTGCG CCGTCATGCG 80
H. bhutanensis  ATGGCCGCTA TCAAGAACAT CACCTCCTCC AAGAACGCCG CCCGCGGCGT CGACCAGTCC AACCCGTGCA ACGTCATGCG 80
H. decipiens    ATGGCCGCTA TCAAGAACAT CACCTCCTCC AAGCACGCCG CCCGCGGCGT CGACCAATCC AACGGATGCT CCGTCATGCG 80
H. savannae     ATGGCCTCTG TCAAGAACAT CACCTCCTCC AAGCACGCCG CCCGCGGCGT CGACCAATCC ACCCCTTGCA GCGTCATGCG 80
H. moniliformis ATGGCCTCCG TCAAGAACAC TACCTCTTCC AAGACTGCCG ACCGTGGCGT CACCCAGGCT CCCCCGTGAA ATGTTATGCG 80
H. fecunda      ATGGCCTCCG TCAAGAACAC TACCTCTTCC AAGACTGCCG ACCGTGGCGT CACCCAGGCT CCCCCGTGAA ATGTTATGCG 80
H. tyalla       ATGGCCTCCG TCAAGAACAC TACCTCTTCC AAGACTGCCG ACCGTGGCGT CACCCAGGCT CCCCCGTGAA ATGTTATGCG 80
```

```
                                  100                   120                   140                   160
                                    |                     |                     |                     |
H. abstrusa     TGGCGTTGAC CAGTCCAGCG GTTGCTCCGT CATGCGCGGT GTTGACCAGT CCAGCGGCTG CACCCTCATG CGCGGTGTTG 160
H. omanensis    CGGCGTTGAC CAGTCCAACC CCTGCGCTGT CATGCGCGGC GTCGACCAGT CCAACCCCTG CACTGTCATG CGCGGTGTTG 160
H. bhutanensis  TGGTGTTGAC CAGTCCAACC CGTGCGCTGT CATGCGCGGT GTTGATCAGT CCAACCCCTG CACCGTCATG ---------- 150
H. decipiens    CGGTGTTGAC CAATCCAACG GTTGCACCGT CATGCGCGGT GTTGACCAGT CCAACGGCTG CACTGTCATG CGCGGTGTTA 160
H. savannae     TGGTGTTGAC CAGTCTACCC CGTGCAACGT CATGCGTGGT GTTGACCAGT CCACCCCGTG CAACGTCATG CGCGGTGTTG 160
H. moniliformis CGGCGTTACC CAGGCCCCGC CTTGCAACGT CATGCGTGGT GTTACCCAGG CTCCTCCCTG CAACGTCATG ---------- 150
H. fecunda      CGGCGTTACC CAGGCCCCGC CTTGCAACGT CATG------ ---------- ---------- ---------- ---------- 114
H. tyalla       CGGCGTTACC CAGGCCCCGC CTTGCAACGT CATGCGCGGC GTTACCCAGG CCCCGCCTTG CAACGTCATG CGTGGTGTTA 160
```

```
                                  180                   200                   220
                                    |                     |                     |
H. abstrusa     ACCAGTCCAG CGG------- ---------- ---------- ---------T TGCACCCTCA TGTAA 189
H. omanensis    ACCAGTCCAA C--------- ---------- ---------- -------CCG TGCACCCTCA TGTAA 189
H. bhutanensis  ---------- ---------- ---------- ---------- ---------- ---------- --TAA 153
H. decipiens    ACCAGTCCAA CGGCTGCACT GTCATGCGCG GTGTTAACCA GTCCAACGGT TGCACCCTCA TGTAA 225
H. savannae     ACCAGTCCAC CCCTTGCACC G--------- ---------- ---------- -------TCA TGTAA 189
H. moniliformis ---------- ---------- ---------- ---------- ---------- ---------- --TAA 153
H. fecunda      ---------- ---------- ---------- ---------- ---------- ---------- --TAA 117
H. tyalla       CCCAGGCTCC TCCCTGCAAC G--------- ---------- ---------- -------TCA TGTAA 189
```

Fig S3: An alignment of the *a1* a-factor pheromone factor genes from all eight *Huntiella* species considered in this study. All three unisexual *Huntiella* species harboured a mutation that converted the TGC codon (Cys) into the TGA stop codon. This alignment was produced using the CLC Main Workbench V22.0 Create Alignment function within the Alignments and Trees module. The parameters were all set to default.

FIGURE S4

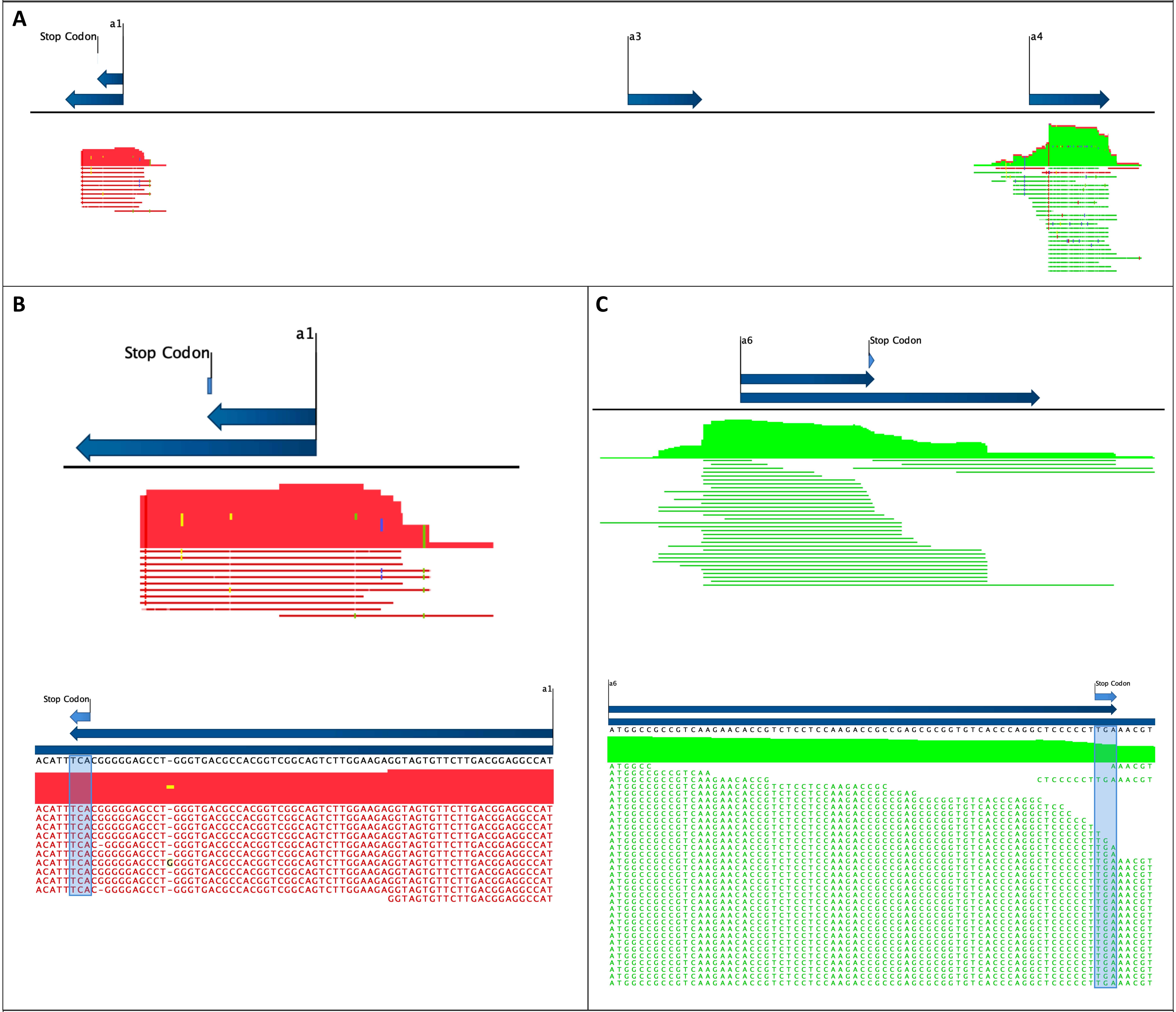

Fig S4: RNA mapping to confirm in frame stop codons in the *a1* and *a6* a-factor pheromone factor genes from the unisexual *H. moniliformis*. A) Overview of the mapping across the first *H. moniliformis* a-factor pheromone locus (*a1, a3* and *a4*). Both *a1* and *a4* are expressed, while there is no evidence that the *a3* gene is expressed. B) Mapping of the *a1* gene, indicating the start codon as well as the in frame stop codon (indicated by the light blue arrow and box). C) Mapping of the *a6* gene, indicating the start codon as well as the in frame stop codon (indicated by the light blue arrow and box). The mappings were produced using the CLC Genomics Workbench V22.0 Map Reads to Contigs function within the De Novo Sequencing module. The parameters were all set to default, except the minimum length and similarity fractions. These values were set to 0.9 for the mappings in A and B, and were set to 1.0 in the mapping in C.

FIGURE S5

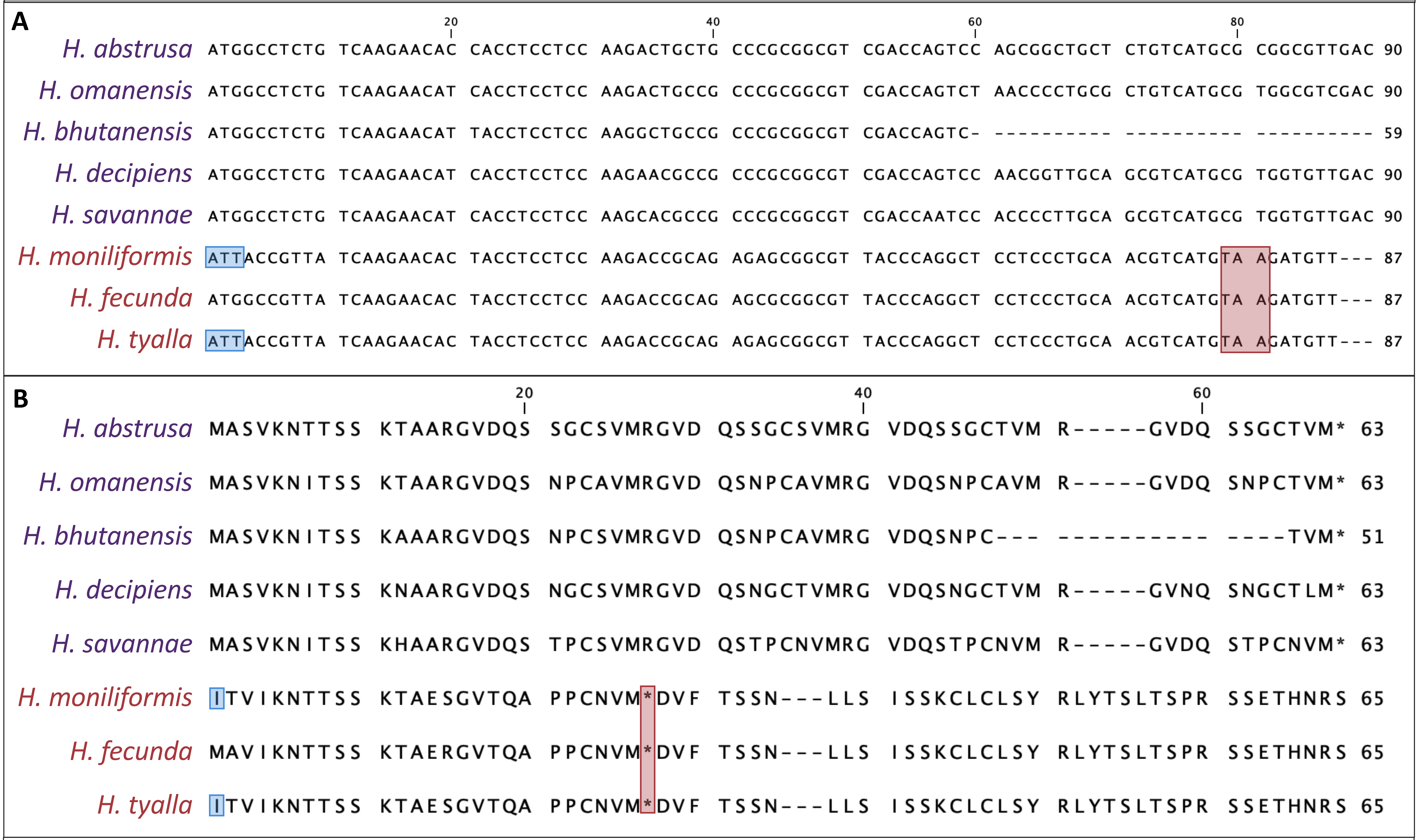

Fig S5: An alignment of the *a3* a-factor pheromone from all eight *Huntiella* species considered in this study. All three unisexual species harboured a stop codon at the position corresponding to the 27th amino acid of the protein, a codon not present in the heterothallic species (indicated by the red blocks). Additionally, *H. moniliformis* and *H. tyalla* also harboured a mutation that converted the ATG codon (start methionine) into an ATT codon (Ile), potentially producing an untranslatable mRNA (indicated by the light blue blocks). A) A gene alignment of the first 90 nucleotides of the *a3* a-factor pheromone gene. B) A protein alignment of the full-length a3 a-factor pheromone protein. This alignment was produced using the CLC Main Workbench V22.0 Create Alignment function within the Alignments and Trees module. The parameters were all set to default.

FIGURE S6

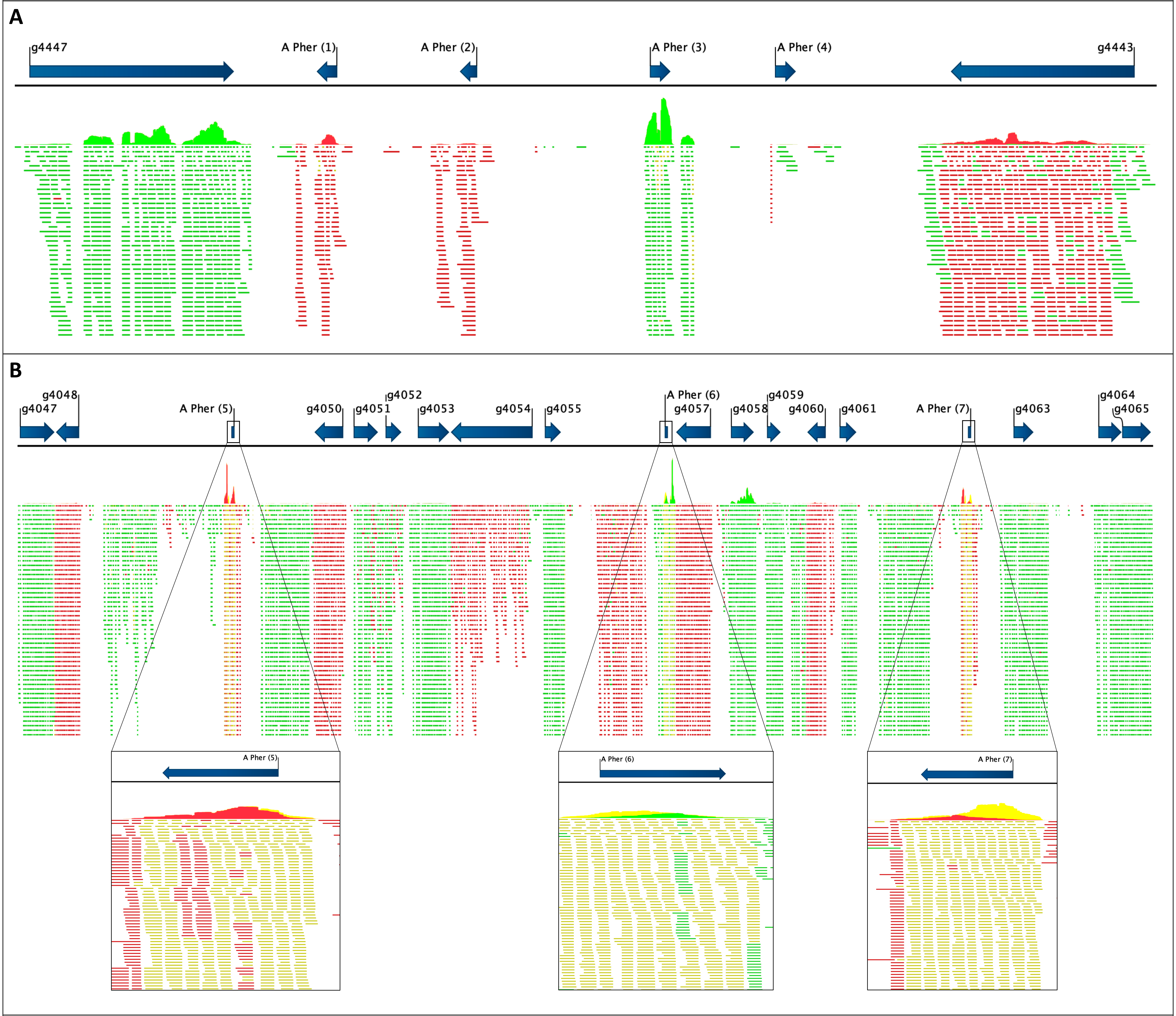

Fig S6: RNA mapping to determine expression of the multiple a-factor pheromone genes from *H. abstrusa*. A) Expression of the genes present at the first a-factor pheromone locus. B) Expression of the genes present at the second a-factor pheromone locus, with inserts zoomed in on the three a-factor pheromones. The mappings were produced using the CLC Genomics Workbench V22.0 Map Reads to Contigs function within the De Novo Sequencing module. The parameters were all set to default, except the minimum length and similarity fractions. These values were both set to 1.0.

FIGURE S7

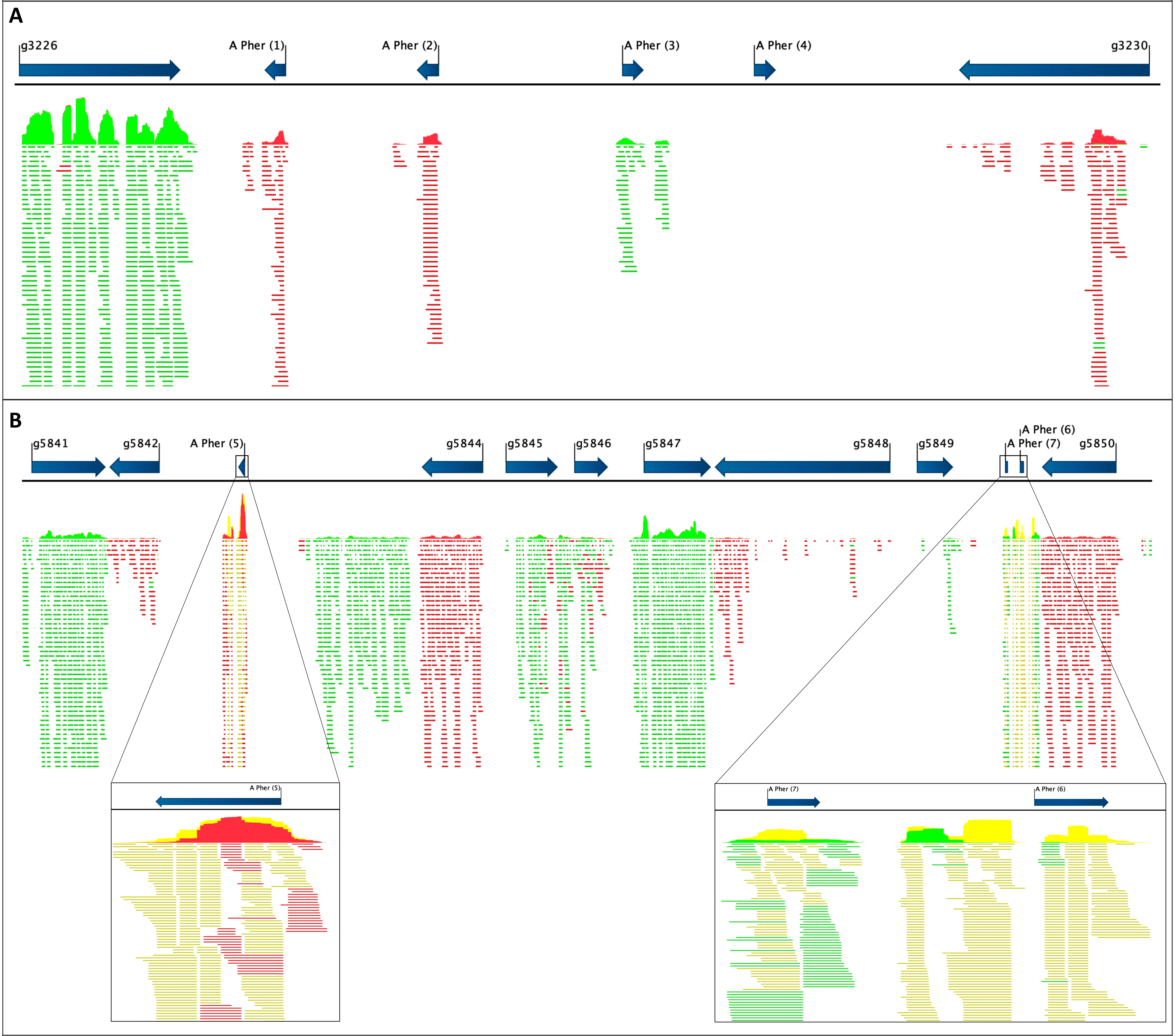

Fig S7: RNA mapping to determine expression of the multiple a-factor pheromone genes from *H. omanensis*. A) Expression of the genes present at the first a-factor pheromone locus. B) Expression of the genes present at the second a-factor pheromone locus, with inserts zoomed in on the three a-factor pheromones. The mappings were produced using the CLC Genomics Workbench V22.0 Map Reads to Contigs function within the De Novo Sequencing module. The parameters were all set to default, except the minimum length and similarity fractions. These values were both set to 1.0.

FIGURE S8

A

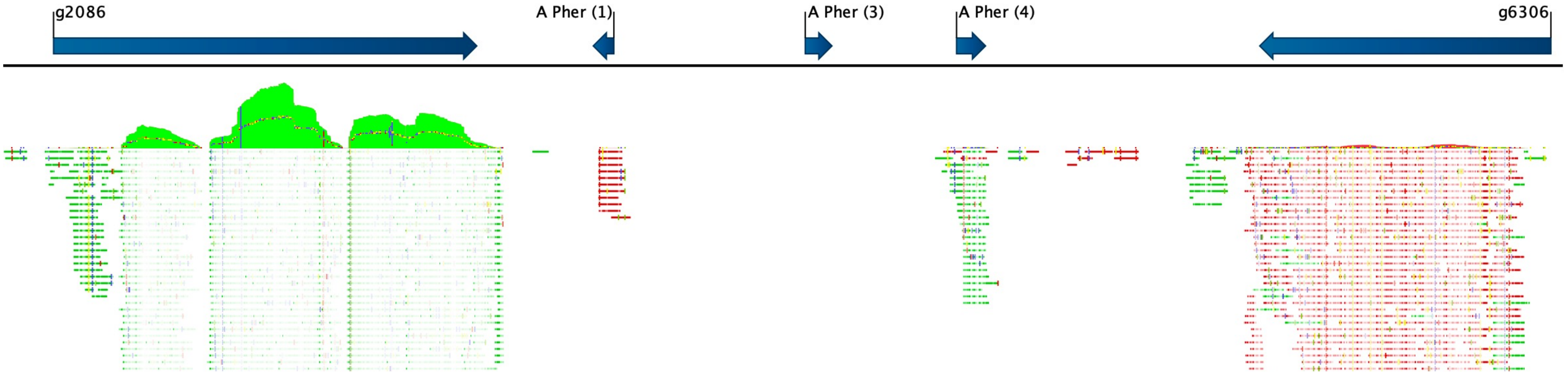

B

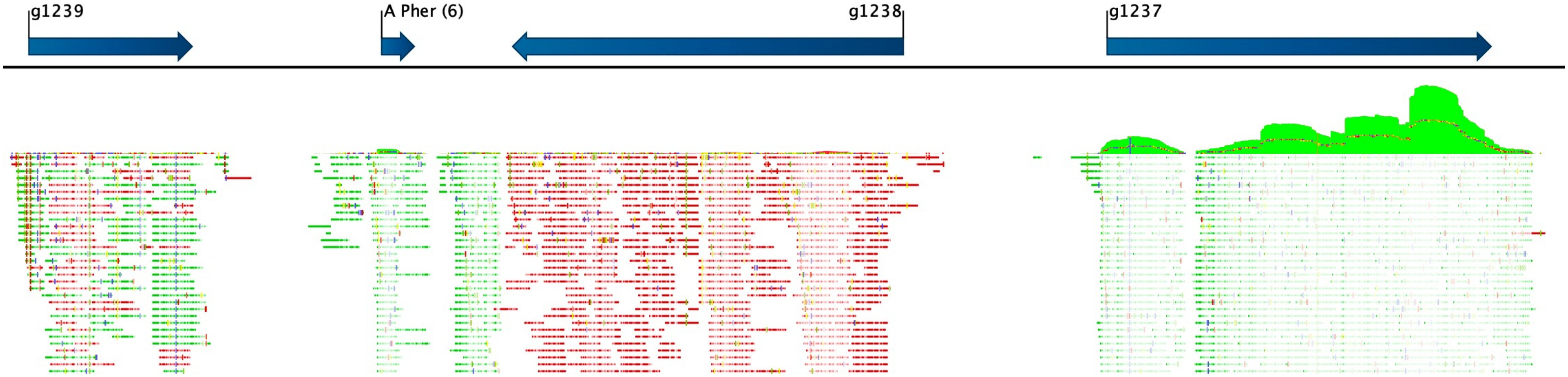

Fig S8: RNA mapping to determine expression of the multiple a-factor pheromone genes from *H. moniliformis*. A) Expression of the genes present at the first a-factor pheromone locus. B) Expression of the genes present at the second a-factor pheromone locus. The mappings were produced using the CLC Genomics Workbench V22.0 Map Reads to Contigs function within the De Novo Sequencing module. The parameters were all set to default, except the minimum length and similarity fractions. These values were set to 1.0 and 0.9, respectively.

FIGURE S9			
**Species**	**Protein structure**	**Repeat sequences**	**Number of repeats**
H. abstrusa	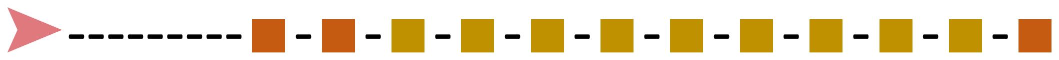	NSNGGLPGELL<br>YSNGGLPGELL	12
H. omanensis	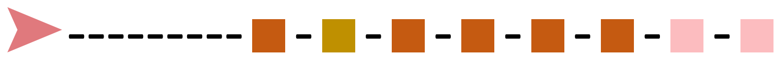	DSNGGLPGELL<br>NSNAGLPGELL<br>YSNAGLPGELL	8
H. bhutanensis	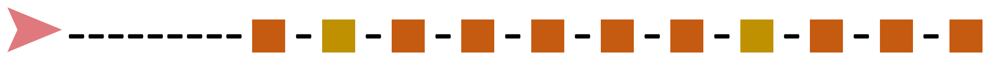	NSNGGLPGELL<br>DSNGGLPGELL	11
H. decipiens	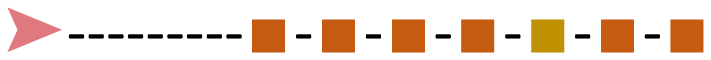	NSNGGLPGELL<br>DSNGGLPGELL	7
H. savannae	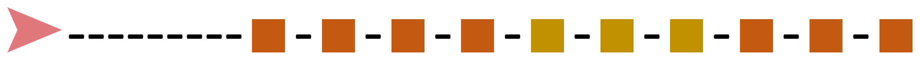	NSNGGLPGELL<br>DSNGGLPGELL	10
H. moniliformis	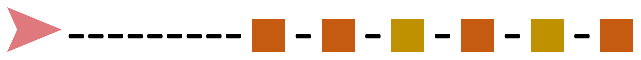	DANGGLPGELF<br>DAWGGLPGELF	6
H. fecunda	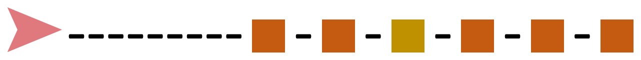	DANGGLPGELF<br>DAWGGLPGELF	6
*H. tyalla**	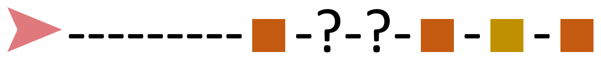	DANGGLPGELF<br>DAWGGLPGELF	6

Fig S9: The structure of the *Huntiella* α-factor pheromones from the various *Huntiella* species along with the sequences of the putative mature repeats. The signal peptide is represented by the light pink triangle and the repeat units are represented by coloured squares. The structure schematics are not drawn to scale. * The *H. tyalla* α-pheromone contig could not be fully assembled.
